# The heart as an endocrine organ

**DOI:** 10.1530/EC-14-0012

**Published:** 2014-04-15

**Authors:** Tsuneo Ogawa, Adolfo J de Bold

**Affiliations:** 1 Cardiovascular Endocrinology Laboratory University of Ottawa Heart Institute 40 Ruskin Street, Ottawa, Ontario, K1Y 4W7 Canada

## Abstract

The concept of the heart as an endocrine organ arises from the observation that the atrial cardiomyocytes in the mammalian heart display a phenotype that is partly that of endocrine cells. Investigations carried out between 1971 and 1983 characterised, by virtue of its natriuretic properties, a polypeptide referred to atrial natriuretic factor (ANF). Another polypeptide isolated from brain in 1988, brain natriuretic peptide (BNP), was subsequently characterised as a second hormone produced by the mammalian heart atria. These peptides were associated with the maintenance of extracellular fluid volume and blood pressure. Later work demonstrated a plethora of other properties for ANF and BNP, now designated cardiac natriuretic peptides (cNPs). In addition to the cNPs, other polypeptide hormones are expressed in the heart that likely act upon the myocardium in a paracrine or autocrine fashion. These include the C-type natriuretic peptide, adrenomedullin, proadrenomedullin N-terminal peptide and endothelin-1. Expression and secretion of ANF and BNP are increased in various cardiovascular pathologies and their levels in blood are used in the diagnosis and prognosis of cardiovascular disease. In addition, therapeutic uses for these peptides or related substances have been found. In all, the discovery of the endocrine heart provided a shift from the classical functional paradigm of the heart that regarded this organ solely as a blood pump to one that regards this organ as self-regulating its workload humorally and that also influences the function of several other organs that control cardiovascular function.

## Historical context

**Figure 1:**
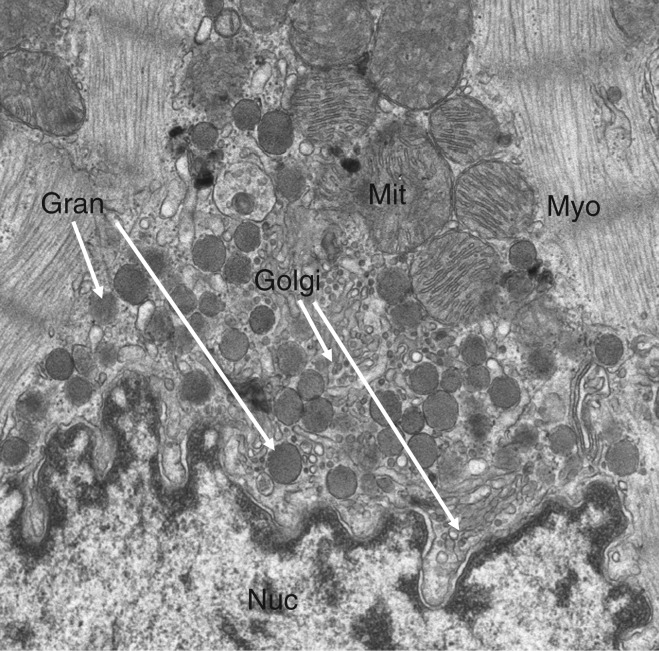
Transmission electron microscopy of a portion of a mouse atrial cardiomyocyte showing a portion of the nucleus (Nuc) as well as mitochondria (Mit), myofibrils (Myo), Golgi complex (Golgi) and specific atrial granules (Gran). Original magnification: 5000×.

The phenotype of ventricular cardiomyocytes reflects functions associated with properties inherent to their mechanical task and the conduction of electrical excitation arising from the sino-atrial node. The advent of the electron microscope demonstrated in mammalian atrial cardiomyocytes elements that were previously associated with polypeptide hormone-producing cells. These included abundant rough endoplasmic reticulum, highly developed Golgi complex and storage granules referred to as atrial-specific granules (Fig. 1) (1, 2).

The function of these differentiations was not evident and so these findings remained functionally an enigma for 2 decades. In 1981, after developing a number of investigative techniques (3, 4, 5, 6, 7, 8), de Bold and colleagues demonstrated that injection of atrial muscle extracts to bioassay rats produced a strong natriuresis and diuresis as well as hypotension accompanied by an increase in haematocrit (9). Subsequently, in 1983, a polypeptide named atrial natriuretic factor (ANF) or atrial natriuretic peptide (ANP) was isolated, purified and sequenced from rat atrial extracts (10, 11, 12). The following year, ANF was isolated from human atria (13). The association of ANF with the atrial-specific granules was demonstrated by both tissue fractionation studies and by immunocytochemistry (14, 15). The presence of natriuretic activity in the atria of mammals and in both the atria and ventricles of non-mammalian vertebrates was subsequently demonstrated corresponding with the morphological distribution of atrial specific-like granules in various species (16). These early findings provided the basis to establish the heart as an endocrine organ. Following the discovery of ANF, related peptides such as brain natriuretic peptide (BNP) and C-type natriuretic peptide (CNP) were described thus establishing a natriuretic peptide (NP) family. ANF and BNP are mainly produced in the heart and so they are referred to as cardiac natriuretic peptides (cNPs). Other unrelated peptide hormones such as the calcitonin gene-related peptide (CGRP) family and endothelin-1 (ET1) are also expressed in the heart albeit in quantities that are smaller than in their principal sites of synthesis. Unlike the cNPs that are truly endocrine products, these hormones may act as cardiac paracrine or autocrine agents.

## The natriuretic peptide family (ANF, BNP and CNP)

**Figure 2:**
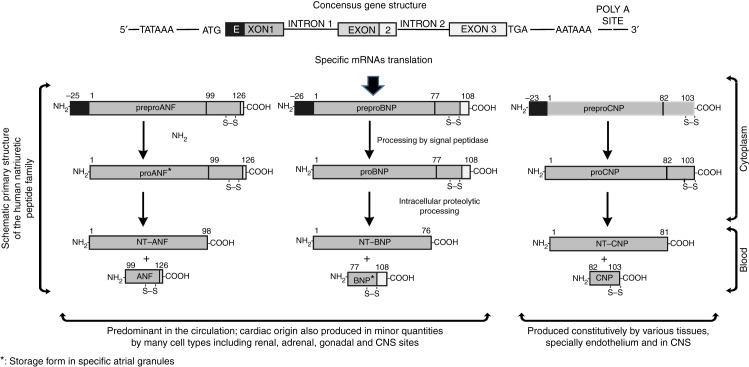
Consensus structure of the natriuretic peptide genes and processing of peptides following translation of the specific mRNAs.

The NPs share a 17 amino acid ring structure formed by an intra-molecular disulphide linkage (Fig. 2, Table 1). This ring structure is essential for biological activity (17, 18). BNP was isolated from porcine brain in 1988. It is a 32-amino acid peptide in humans (18). Although originally isolated from porcine brain, BNP is most abundantly expressed in the atria of the heart. ANF and BNP are functionally considered to be the main endocrine products of the heart. ANF and BNP are co-stored in atrial-specific granules (15, 19). A third ‘natriuretic’ peptide, CNP, is a 22-amino acid peptide isolated from porcine brain in 1990. It lacks the carboxyl terminal of ANF and BNP and hence it lacks natriuretic properties (20). CNP is produced in a constitutive manner in the brain and pituitary gland, and also in the endothelium, smooth muscle cells of the intima, media and vasa vasorum of arteries and in macrophages in normal and atheromatous coronary arteries in humans (20, 21, 22). Although initial investigations failed to find CNP in the heart (23), its presence was subsequently shown by RIA, immunocytochemistry and RT-PCR (24). The C-terminal portions of human ANF and BNP: ANF_99–126_ and BNP_77–108_ are the biologically activepeptides derived from processing proANF and proBNP by cardiomyocytes before release into the circulation.

Plasma levels of ANF and BNP in normal subjects are 3.2–19.5 and 1.4–14.5 pmol/l respectively (25, 26). The greater part of ANF and BNP gene expression in mammals is found in the atria of the heart. Other sites, such as the cardiac ventricles, aortic arch as well as extra cardiac sites including hypothalamus, pituitary gland and lung also produce cNPs at much lower levels than the atria (27, 28, 29).

Although it is often mentioned that BNP is a ventricular hormone while ANF is viewed as an atrial hormone, this concept needs to be reconsidered. In certain pathophysiological conditions, ANF and BNP plasma levels increase significantly and while ANF remains higher than BNP, the latter increases only relatively more than ANF (30, 31, 32).

The biochemical evidence supports an overwhelming atrial source of cNPs. The ANF concentration in normal subjects in atrium and ventricle is 9600 and 37 pmol/g respectively, while the BNP concentration of normal subjects in atrium and ventricle is 250 and 18 pmol/g respectively (33). Even taking into account the differences in mass between the atrial and ventricular chambers, it is not possible to conclude that BNP is a ventricular hormone. Similar conclusions can be reached when evaluating transcript and peptide abundance for atrial and ventricular ANF and BNP (32, 34).

Cardiac catheterisation data in humans with left ventricular hypertrophy showed that the abnormal circulating levels of cNPs are significantly derived from atrial sources (35). Replacement of the failing ventricle in orthotropic heart transplantation in human does not result in normalisation of either BNP or ANF plasma levels even after normalisation of the intracardiac pressures and the renin–angiotensin–aldosterone system (30). From the pathophysiological point of view, these findings show that while there may be a good correlation between parameters, such as ventricular hypertrophy or failure or ischemic events and cNPs secretion, this secretion may not be considered of ventricular origin.

Both ANF and BNP are synthesised in and secreted from the atria in a regulated manner. In ventricles, given the practical absence of granules in the bulk of the muscle, the minute amounts secreted are said to occur via the constitutive pathway (19). However, this has never been formally demonstrated.

ANF and BNP are continuously secreted from the atria under basal conditions. Mechanical stretch of atrial muscle increases the rate of peptide secretion rapidly followed by a sharp decline to baseline. This transient increased secretion of ANF and BNP is not due to the cNPs pool depletion but due to the decreased sensitivity to the stimulating signal for regulated release (36). ET1 and phenylephrine (PE) also increase ANF and BNP secretion, but the rate of increase and decline in the secretion are more gradual compared with mechanical stretch (37, 38, 39). The protein synthesis inhibitor cycloheximide partially but not completely abolishes basal cNP secretion (36), but Monensin, an ionophore that impairs protein sorting and transport in the trans-Golgi network and that inhibits vesicle formation without directly affecting protein synthesis, completely inhibited stimulated secretion without affecting baseline release. Double-label pulse-chase analysis showed that cardiomyocytes preferentially release newly synthesised hormone in a manner that does not entirely rely on protein synthesis (40). These findings suggest that a constitutive-like release, which is based on an exocytosis of vesicles budding from immature granules, is the main release mechanism of operation in atrial cardiomyocytes. Constitutive-like release is independent of the regulated and constitutive pathways (41).

Many of the agonists of cNPs secretion, such as ET1, angiotensin II (AII) and PE, signal through the receptors coupled to Gq proteins, although other subclasses may also be used (42, 43) and stimulate the RAS/c-Raf-1 and the phosphoinositide signalling pathways leading to the recruitment and activation of effector molecules such as those found in MAP kinase cascades (44, 45). Gi/o inhibition by pertussis toxin (PTX) abolishes stretch-secretion coupling without affecting baseline secretion through a mechanism that is independent of Gq signalling, suggesting that PTX sensitive Gi/o proteins are involved in stretch-induced secretion in atria. By immunocytochemistry, ANF and Gαo are seen co-localised in atrial granules, suggesting a role for this G protein in the secretion of ANF and its sensitivity to PTX (46).

An involvement of glucagon-like peptide-1 (GLP1) has recently been implicated in the regulation of ANF secretion from the heart. GLP1 is one of the incretin hormones secreted from endocrine cells located in the epithelium of the small intestine. Glucose-stimulated incretin is carried through the circulation to the pancreatic β cells where it stimulates insulin secretion. Blood pressure reduction has been reported in type-2 diabetic patients treated with GLP1 analogues or dipeptidyl peptidase-4 enzyme inhibitors (47, 48). Gene expression of the cardiac GLP1 receptor was detected in atria but not in ventricles. GLP1 receptor activation promoted ANF secretion and blood pressure reduction. These effects were not seen in GLP1 receptor knockout mice or ANF knockout mice (49). These data suggest that an increase in ANF secretion from the atria is at least partly involved in the antihypertensive effect of GLP1 receptor agonists.

The biological effects of ANF and BNP are predominantly mediated through the NPR-A receptor, a guanylyl cyclase-coupled receptor, that is widely distributed throughout the body, including the kidneys, vascular smooth muscle, adrenals, brain and heart (50, 51). NPR-B, a second guanylyl cyclase-coupled receptor, is associated with CNP signalling. Five domains are recognised in NPR-A and NPR-B: an extracellular ligand-binding domain that binds NP, a short transmembrane domain, a kinase homology domain, a dimerisation domain and a guanylyl cyclase domain (52). Binding of agonists to the NP receptors results in an increase in intracellular cGMP that can spill over to the general circulation. Systemic administration of ANF elevates urinary and circulating levels of cGMP (53).

Intracellular cGMP targets several enzymes and ion channels including cGMP-dependent protein kinases, cGMP-gated ion channels and cGMP-regulated cyclic nucleotide phosphodiesterases (54). Changes in the activity of these enzymes or channels result in a vasorelaxant effect, inhibition of Na^+^ reabsorption in renal inner medullary collecting ducts and in various other effects such as modulation of steroidogenesis in the adrenal glands and phototransduction (55, 56).

A third natriuretic peptide receptor, NPR-C, is considered to be a clearance receptor, although G-protein signalling has been associated with it (57). The short 37-amino acid cytoplasmic segment of NPR-C is characteristic of several receptors involved in peptide and macromolecule clearance. NPR-C is found most abundantly in the glomerular and vascular structures of the kidney, and also in the adrenals, lungs, brain, heart and the vascular wall (58, 59).

The binding potency of the NPRs is ranked as follows: NPR-A=ANF≥BNP>CNP; NPR-B=CNP≥ANF>BNP; NPR-C=ANF≥CNP>BNP (60).

From the haemodynamic point of view the endocrine heart serves to regulate cardiac preload and afterload due to its influence in the regulation of extracellular fluid volume mediated by targets such as the kidney, the renin–angiotensin–aldosterone system and arterial and sympathetic tones. However, many other targets have been characterised (for a review see 61, 62, 63).

In the kidneys ANF and BNP increase glomerular infiltration rate and filtration fraction by dilating afferent arterioles and constricting efferent arterioles leading to an increase in glomerular capillary hydraulic pressure that enhances the driving force for ultrafiltration. ANF and BNP also inhibit Na^+^ reabsorption at the level of the renal collecting duct (64, 65). These renal functions of ANF and BNP underlie their acute strong diuretic and natriuretic effects.

ANF and BNP have multiple actions on nerve activity. ANF reduces cardiac and pulmonary chemo- and baroreceptor activity, which leads to the suppression of sympathetic outflow to the heart. A decrease in sympathetic activity together with the increase in vagal afferent activity leads to a reduction in heart rate and cardiac output (66). ANF and BNP reduce vascular smooth muscle tone and peripheral vascular resistance and also decrease salt and water appetite, thus leading to a reduction in extracellular fluid volume (67). ANF inhibits secretion of vasopressin from the posterior pituitary leading to decreases in water reabsorption in the collecting duct of the kidney (68).

The cNPs possess growth-suppressing and anti-proliferative properties. Growth promoting effects generated by norepinephrine are inhibited by ANF treatment on both cardiomyocytes and fibroblasts (69). BNP inhibits cardiac intersitium remodelling through its antifibrotic effects (70) and also inhibits transforming growth factor-induced fibrosis in association with cGMP-dependent protein kinase and the MEK/ERK pathway (71). ANF antagonises the growth of vascular smooth muscle cells (72) and promotes endothelial cell function in atherosclerosis (73).

cNPs antagonise the renin–angiotensin–aldosterone system by inhibiting aldosterone synthesis and secretion, and by suppressing renin release (74, 75, 76).

cNPs exert potent lipolytic effects in isolated human fat cells as well as in preadipocytes through cGMP-mediated phosphorylation (77, 78, 79, 80, 81). Cardiac cachexia often seen in the heart failure, during which ANF and BNP are increased, may be partly explained by the lipolytic effects of cNPs. Finally, ANF inhibits the proliferation of human visceral preadipocytes (82).

A change in haemodynamic load or neurohumoral stimulation can lead to increase cNPs synthesis and/or secretion. The endocrine heart responds differently to haemodynamic loads, depending on whether the challenge is acute (hours), subacute (24 h to 1 week) or chronic (longer than a week).

The increased secretion of cNPs following acute mechanical atrial stretch is based on a phenomenon referred to as stretch-secretion coupling (83). This event is characterised by a phasic, short-term burst of cNPs secretion with no apparent effect on synthesis (44). In stretch-secretion coupling, the predominant circulating cNPs source is the existing atrial NPs pool.

Subacute haemodynamic load is observed during mineralocorticoid escape, which is characterised by a transient period of positive sodium balance resulting from chronic exposure to mineralocorticoid excess followed by a vigorous natriuresis leading to a new steady-state of sodium balance. The increase in intravenous volume and central venous pressure leads to increased ANF and BNP production in atria but not in ventricles, in which plasma ANF but not BNP is significantly elevated. In subacute haemodynamic load, the predominant circulating cNP source is a stored hormone plus newly synthesised hormone (44).

Both, atria and ventricles increase synthesis and secretion of ANF and BNP during chronic haemodynamic overload. This condition is seen in chronic arterial hypertension and chronic congestive heart failure and is accompanied by the ventricular re-expression of the cardiac foetal gene programme, which includes the re-expression of both ANF and BNP (28, 32). The main source of cNPs, however, remains the cardiac atria, despite a significant increase in the cNPs content in the ventricles is also observed.

In addition to mechanical stimuli, circulating neuroendocrine agents can act on atrial cardiomyocytes to modulate cNP synthesis and secretion. These include ET1, hydroxyvitamin D_3_, glucocorticoids, thyroid hormones, several growth factors, thrombin, AII, prostaglandins and α_1_-adrenergic agents (34, 84, 85, 86, 87). The proinflammatory cytokines such as TNFα and IL1β and lipopolysaccharide stimulate the synthesis and secretion of BNP specifically (88, 89).

cNPs blood levels are increased in various pathological conditions such as heart failure, myocardial infarction, hypertension, left ventricular hypertrophy and pulmonary hypertension (90, 91, 92, 93, 94, 95, 96, 97, 98). Determination of these levels is useful in the diagnosis and prognosis of these cardiovascular pathologies. In addition to ANF_99–126_ and BNP_77–108_, the biologically inactive N-terminal fragments of the cNP prohormones, NT-proANF and NT-proBNP, also circulate in plasma. As these N-terminal fragments are more stable in the circulation and their half-lives are longer than those of the C-terminal prohormones (99, 100) the usefulness of NT-proANF and NT-proBNP as biomarkers of disease has been extensively investigated. BNP has been found to perform somewhat better than ANF and NT-proANF (101, 102, 103) in diagnosis and prognosis. NT-proBNP and BNP provide interchangeable information, although NT-proBNP seems to perform better in diagnosing mild heart failure or asymptomatic left ventricular dysfunction (104). At present BNP and NT-proBNP are established diagnostic biomarkers for heart failure (105, 106), ventricular remodelling after acute myocardial infarction (96, 107), left ventricular hypertrophy (103, 108) and pulmonary hypertension (109).

An immunoassay that detects the midregional fragment of proANF (MR-proANP) has been developed (110). In a study in 797 chronic congenital heart failure patients, MR-proANP outperformed BNP and NT-proBNP in the prediction of death (111). In another study of 251 patients with dyspnoea, BNP and MR-proANP provided similar diagnostic information and were clinically useful as an aid in the diagnosis of acutely decompensated heart failure (112).

Because the cNPs possess diuretic, natriuretic and hypotensive activity, ANF and BNP have been used in the treatment of various cardiovascular conditions. As ANP and BNP bind to the same receptor, NPR-A, it would be expected that the therapeutic effects of these peptides would be similar, but some differences between ANF and BNP are apparent in the clinical settings. The infusion of carperitide, a recombinant form of human ANF, improved congestive heart failure and acute myocardial infarction, and also had renal protective effects on contrast-induced nephropathy (113, 114, 115, 116). However, the effect and safety of nesiritide, a recombinant form of BNP, has been questioned. According to one study, the infusion of nesiritide to heart failure patients increased the risk of worsening renal function (117). Other trials with nesiritide in heart failure patients did not show any changes regarding death rate, re-hospitalisation rate and renal function, and hence the routine use of nesiritide to acute failure patients was not recommended (118). In another study, s.c. administration of BNP twice a day for 8 weeks improved haemodynamics in heart failure patients with preserved glomerular filtration rate (119).

The third member of NP family, CNP, is produced within the heart in a constitutive-like manner. Plasma CNP levels are 1–6 pmol/l, which is one-third of those for ANF (120, 121). High concentrations of CNP are found in the CNS, especially in the pituitary gland (21). Relatively low levels of CNP are found in kidneys, bone, blood vessels and heart (122). The CNP concentration in atrium is 0.11±0.018 pmol/g, which is 100 times less than that found in the CNS (120). In the cardiovascular system, *CNP* gene expression is found mainly in endothelial cells and to some extent in cardiomyocytes and cardiac fibroblasts (24, 123, 124, 125). Blood samples taken during cardiac catheterisation in patients with congestive heart failure, show that *CNP* plasma levels in the coronary sinus is significantly higher than that in aorta, suggesting that *CNP* is synthesised in the human heart (125, 126). *CNP* secretion from endothelial cells is stimulated by various cytokines, such as transforming growth factor, tumour necrosis factor and interleukin-1 (123, 127). ANF and BNP also stimulate the synthesis and secretion of *CNP* in endothelial cells (128).

CNP possesses potent vasodilating properties on isolated blood vessels and produces growth inhibition of vascular smooth muscle cells (129, 130). *NPR-B* gene expression is observed in vascular smooth muscle, suggesting that CNP acts locally to inhibit vasoconstriction and vascular growth. CNP also has direct effects on cardiac function where it acts initially as a positive inotropic and lusitropic agent followed by a negative inotropic effect in the isolated heart (131, 132, 133). CNP inhibits ET1-induced cardiomyocyte hypertrophy via a cGMP-dependent mechanism (134) as well as interstitial fibrosis and collagen I and III gene expression in chronically Ang II-infused mice (135).

I.v. administration of CNP to healthy volunteers decreased blood pressure with an increase in diuretic and natriuretic activities in doses ten times higher than those used to compare it with ANF (136). In congestive heart failure, plasma levels and atrial concentration of CNP are increased (121, 137). It is assumed that in volume overload, the increased ANF and BNP together with the pro-inflammatory cytokines stimulate the synthesis of CNP in heart, which in turn exerts local vasodilation and inhibits vascular growth and cardiac hypertrophy.

## Adrenomedullin and related peptides

Adrenomedullin (AM) is a 52-amino acid peptide first isolated from human pheochromocytoma in 1993 (138). The structure of AM makes it a member of the calcitonin superfamily, which includes CGRP, calcitonin and amylin. AM contains a six-amino acid ring structure formed by disulphide bond and an amidated C-terminal tyrosine residue, both of which are essential for biological activity.

A unique 20 residue sequence followed by Gly-Lys-Arg, a typical amidation signal, exists in the N-terminal region of preproadrenomedullin that was found in human pheochromocytoma (139). This peptide was named proadrenomedullin N-terminal 20 peptide (PAMP) and shows no similarity with any other peptides.

Plasma level of AM is 2–10 pmol/l, which is comparable with ANF plasma levels (140, 141). In human, highest gene expression and highest-immunoreactive *AM* is seen in adrenal medulla, which is more than ten times higher than in ventricle or atria (142). *AM* gene expression is also seen in cardiomyocytes, cardiac fibroblasts, vascular endothelial cells and smooth muscle cells (143, 144, 145). A large part of plasma *AM* originates from the endothelial cells in various organs (146).

*AM* binds to three types of receptors structurally made of two components: one is a calcitonin receptor-like receptor and the other one is denominated receptor activity-modifying protein (147). These receptors are coupled to G protein and adenylyl cyclase.

AM possesses a wide spectrum of biological actions such as vasodilation, natriuresis and diuresis (148) as well as the inhibition of proliferation of cardiac fibroblasts and the production of extracellular matrix (149).

Plasma AM is increased in various pathological condition such as essential hypertension, acute coronary syndrome, congestive heart failure and septic shock (150, 151, 152, 153, 154). The increased production of cardiac AM is believed to have a cardio protective effect by decreasing pressure overload, cardiac mass as well as fibrosis.

Although both the cNPs and AM possess hypotensive, diuretic and natriuretic properties, the precise regulation of circulating levels of these peptides might be different because cNPs are synthesised mainly in heart, whereas the principal production sites of AM are blood vessels and adrenal medulla.

## Proadrenomedullin N-terminal 20 peptide

Plasma level of PAMP is 0.1–1.0 pmol/l, which is 10–20% of plasma AM concentration (139, 141, 155). The highest concentration of PAMP is seen in adrenal medulla at 13.8±7.9 pmol/g. A relatively high concentration of PAMP is detected in atria of the heart and low levels in ventricles, lungs and kidneys (139).

PAMP is a potent hypotensive peptide. The blood pressure lowering mechanism is different from that of AM (156). AM reduces vascular tone while PAMP reduces sympathetic tone (157). Further, PAMP does not affect protein synthesis in cardiomyocytes or cardiac fibroblasts (159).

Similarly to AM, plasma levels of PAMP are increased in patients with a variety of diseases such as essential hypertension, congestive heart failure, chronic renal failure and septic shock (140, 155, 159, 160). This is to be expected given that PAMP and AM are processing products from the same precursor.

## Endothelin-1

The 21-amino acid peptide ET was first isolated from the conditioned medium of cultured endothelial cells and then sequenced in 1988 (161). Shortly after, two structurally similar peptides differing by two and six amino acids were identified. Altogether, the peptides were designated ET1, -2 and -3 respectively (162, 163). ET1 is the predominant isoform and biologically most relevant (164). Plasma level of ET1 is 2–6 pmol/l. The highest concentration of ET1 is found in blood vessels (endothelium) (165, 166). To a lesser extent, ET1 expression is found in other tissues including cardiomyocytes, vascular smooth muscle cells and kidney (167, 168).

ET1 produces a potent and long-lasting vasoconstriction resulting in an increase in blood pressure (161). ET1 also has a direct positive inotropic and chronotropic effects on heart muscle (169). In cultured human cardiac fibroblast, ET1 elicits collagen synthesis. ET1 stimulates the secretion and synthesis of ANF and BNP in heart (39, 170, 171).

There are two types of G-protein-coupled receptors for ET: ETA and ETB (172). ET1 exerts a vasoconstricting and growth-promoting action through the ETA receptor found in smooth muscle cells. Through the ETB receptor located in endothelial cells, ET1elicits vasodilation mediated by nitric oxide (173).

ET1 production is stimulated by hypoxia, angiotensin II, cytokines, growth factors and reduced by nitric oxide and ANF (174, 175, 176, 177, 178, 179, 180). ET1 plasma levels are increased in congestive heart failure, myocardial infarction, pulmonary hypertension and renal failure. Several findings suggest that ET1 produced locally in the heart may have hypertrophic and fibrotic effects, thus contributing to cardiac remodelling (181, 182, 183).

## Conclusion

The heart as an endocrine organ owes its designation to the secretory phenotype of mammalian atrial cardiomyocytes that is associated with the production of the natriuretic polypeptide hormones ANF and BNP. In non-mammalian vertebrates, as well as in the mammalian fetus, the secretory phenotype is displayed by both atrial and ventricular cardiomyocytes. It should be noted that cardiomyocytes in the ventricular conduction system of mammals also display a secretory phenotype.

Cells in the heart also express other polypeptide hormones and their receptors, such as AM and ET1. The expression levels of these hormones in the heart are increased in various cardiovascular pathologies such as hypertension, cardiac hypertrophy, congestive heart failure and acute coronary syndrome. Whether these hormones spill over to contribute to the observed increases in plasma levels is often not clear so that the locally synthesised hormones may be viewed as acting locally within the heart in a paracrine and/or autocrine fashion.

In terms of overall properties of hormones found in the heart, those belonging to the natriuretic peptide family or AM-related peptides have hypotensive, natriuretic and diuretic properties. Many of these peptides also have anti-proliferative and anti-fibrotic properties and thus may be involved in preventing cardiac remodelling. On the other hand, ET1 has hypertensive, volume retentive, and hypertrophic properties that while essential for maintaining cardiovascular homeostasis in cases of massive haemorrhage or hypotensive conditions, can lead to hypertrophic or fibrotic changes in the heart under protracted action.

Regardless of whether their main site of production is cardiac or not, the polypeptide hormones mentioned above appear to contribute cooperatively to regulate cardiovascular haemodynamics through their actions on vascular tone, autonomic nerve activity and cardiac mass in both health and disease.

## Figures and Tables

**Table 1:** Primary structure of various mammalian natriuretic peptides.

**Name**	**Sequence**	**Reference**
ANF		
Rat (all rodents) ANF_99–126_	Ser-Leu-Arg-Arg-Ser-Ser-Cys-Phe-Gly-Gly-Arg-Ile-Asp-Arg-Ile-Gly-Ala-Gln-Ser-Gly-Leu-Gly-Cys-Asn-Ser-Phe-Arg-Tyr	(11)
Human, canine, bovine ANF_99–126_	Ser-Leu-Arg-Arg-Ser-Ser-Cys-Phe-Gly-Gly-Arg-Met-Asp-Arg-Ile-Gly-Ala-Gln-Ser-Gly-Leu-Gly-Cys-Asn-Ser-Phe- Arg-Tyr	(13)
Urodilatin: ANF_99–126_	Thr-Ala-Pro-Arg-Ser-Leu-Arg-Arg-Ser-Ser-Cys-Phe-Gly-Gly-Arg-Met-Asp-Arg-Ile-Gly-Ala-Gln-Ser-Gly-Leu-Gly-Cys-Asn-Ser-Phe- Arg-Tyr	(184)
BNP		
Rat BNP_51–95_	Ser-Gln-Asp-Ser-Ala-Phe-Arg-Ile-Gln-Glu-Arg-Leu-Arg-Asn-Ser-Lys-Met-Ala-His-Ser-Ser-Ser-Cys-Phe-Gly-Gln-Lys-Ile-Asp-Arg-Ile-Gly-Ala-Val-Ser-Arg-Leu-Gly-Cys-Asp-Gly-Leu-Arg-Leu-Phe	(185)
Human BNP_77–108_	Ser-Pro-Lys-Met-Val-Gln-Gly-Ser-Gly-Cys-Phe-Gly-Arg-Lys-Met-Asp-Arg-Ile-Ser-Ser-Ser-Ser-Gly-Leu-Gly-Cys-Lys-Val-Leu-Arg-Arg-His	(186, 187)
Canine BNP_74–105_	Ser-Pro-Lys-Met-Met-His-Lys-Ser-Gly-Cys-Phe-Gly-Arg-Arg-Leu-Asp-Arg-Ile-Gly-Ser-Leu-Ser-Gly-Leu-Gly-Cys-Asn-Val-Leu-Arg-Lys-Tyr	(186)
Mouse BNP_77–121_	Ser-Gln-Gly-Ser-Thr-Leu-Arg-Val-Gln-Gln-Arg-Pro-Gln-Asn-Ser-Lys-Val-Thr-His-Ile-Ser-Ser-Cys-Phe-Gly-His-Lys-Ile-Asp-Arg-Ile-Gly-Ser-Val-Ser-Arg-Leu-Gly-Cys-Asn-Ala-Leu-Lys-Leu-Leu	(188)
Porcine BNP_75–106_	Ser-Pro-Lys-Thr-Met-Arg-Asp-Ser-Gly-Cys-Phe-Gly-Arg-Arg-Leu-Asp-Arg-Ile-Gly-Ser-Leu-Ser-Gly-Leu-Gly-Cys-Asn-Val-Leu-Arg-Arg-Tyr	(18)
CNP		
Human, rat, porcine CNP_82–103_	Gly-Leu-Ser-Lys-Gly-Cys-Phe-Gly-Leu-Lys-Leu-Asp-Arg-Ile-Gly-Ser-Met-Ser-Gly-Leu-Gly-Cys	(20)
Porcine CNP_51–103_	Asp-Leu-Arg-Val-Asp-Thr-Lys-Ser-Arg-Ala-Ala-Trp-Ala-Arg-Leu-Leu-Glu-Glu-His-Pro-Asn-Ala-Arg-Lys-Tyr-Lys-Gly-Ala-Asn-Lys-Lys-Gly-Leu-Ser-Lys-Gly-Cys-Phe-Gly-Leu-Lys-Leu-Asp-Arg-Ile-Gly-Ser-Met-Ser-Gly-Leu-Gly-Cys	(189)
